# A spatially resolved single-cell landscape of colorectal cancer liver metastasis reveals a stromal-tumor glycolytic signaling interaction

**DOI:** 10.3389/fcell.2025.1687485

**Published:** 2025-10-29

**Authors:** Jiahui Chen, Zukai Wang, Bingwang Zhu, Guoxian Guan

**Affiliations:** ^1^ Department of Colorectal Surgery, The First Affiliated Hospital of Fujian Medical University, Fujian Medical University, Fuzhou, China; ^2^ Department of Colorectal Surgery, National Regional Medical Center, Binhai Campus of the First Affiliated Hospital, Fujian Medical University, Fuzhou, China

**Keywords:** colorectal cancer liver metastasis, single-cell and spatial transcriptomics, high-malignancy CRC subpopulation, HGF-MET-MYC signaling axis, spatial stromal-tumor co-localization

## Abstract

**Background:**

Colorectal cancer (CRC) remains a leading cause of cancer mortality, with liver metastasis being the principal determinant of poor prognosis, but the spatial mechanisms orchestrating metastatic niches remain elusive.

**Method:**

To dissect the molecular and spatial dynamics of CRC progression, we constructed an integrative atlas using 35 single-cell RNA-seq datasets and spatial transcriptomics from primary tumors, liver metastases, and matched normal tissues. Malignant epithelial subpopulations were stratified via inferCNV and CytoTRACE analyses. Stromal-tumor interactions were dissected using CellChat and NicheNet, with functional validation through *in vitro* co-culture and immunohistochemistry.

**Result:**

We identified a transcriptionally distinct epithelial subpopulation, termed high-malignancy CRC (High-M CRC), enriched in metastatic lesions and characterized by enhanced stemness, MYC-driven transcriptional activity, and glycolytic reprogramming. Stromal-tumor interaction analyses revealed that cancer-associated fibroblasts (CAFs), particularly matrix CAFs (mCAFs), promote malignant progression via the HGF-MET-MYC signaling axis. Spatial transcriptomic mapping confirmed the physical proximity and molecular co-localization of High-M CRC cells and mCAFs, along with enriched glycolysis and MYC expression at the cell-cell interface. *In vitro* functional validation demonstrated that CAF-derived HGF activates MET-MYC signaling in CRC cells, enhancing their invasion and proliferation—effects reversible by MET knockdown.

**Conclusion:**

We unveil a spatially organized metabolic niche driven by stromal-tumor HGF-MET-MYC signaling. These findings offer novel insights into the stromal-tumor interaction and suggest actionable targets for therapeutic intervention in CRC.

## Introduction

Colorectal cancer (CRC) is the third most commonly diagnosed malignancy and the second leading cause of cancer-related mortality worldwide, with over 1.9 million new cases and nearly 935,000 estimated deaths annually (Matsuda et al., 2025). Metastasis, particularly to the liver, remains the primary determinant of CRC prognosis, with over 50% of patients developing liver metastases (CRLM) during the disease progression. Despite advances in systemic therapies, the 5-year survival rate for metastatic CRC (mCRC) remains below 20% (Zhou et al., 2022). Over the past decade, research has increasingly focused on the role of the tumor microenvironment (TME) in supporting metastatic progression, including the interaction between cancer cells, immune populations, stromal components, and their dynamic reprogramming during dissemination (Chu et al., 2024; Almusawi et al., 2021). However, traditional bulk transcriptomics often mask the cellular heterogeneity inherent to CRC progression and fail to capture the intercellular communication which is critical to metastatic colonization.

The advent of single-cell RNA sequencing (scRNA-seq) and spatial transcriptomics (ST) technologies has revolutionized cancer research by allowing high-resolution dissection of tumor ecosystems. These approaches enable the precise deconvolution of heterogeneous cellular populations, identification of rare or transitional phenotypes, and characterization of cell-specific gene expression profiles within their native spatial contexts (Jain and Eadon, 2024; Lei et al., 2021). In CRC, scRNA-seq has been employed to reveal hierarchies of stem-like tumor epithelial cells, immune evasion mechanisms, and the immunosuppressive landscape of the TME (Wang et al., 2023). For instance, Lin et al. identified distinct transcriptional states of tumor epithelial cells with different activity of cancer stemness, while Chu et al. mapped diverse T cell phenotypes and the immune evasion mechanisms in CRC tumors, highlighting immune cell dysfunction in tumors (Chu et al., 2024; Lin et al., 2024). Beyond epithelial and immune cells, a growing body of work has underscored the central role of stromal components, particularly cancer-associated fibroblasts (CAFs), in shaping tumor behavior (Kobayashi et al., 2022).

CAFs, a functionally diverse and phenotypically plastic population of stromal cells, are now recognized as key components of the TME. Emerging evidence highlights the active role of CAFs in tumorigenesis. Abundant scRNA-seq studies across multiple cancer types, including pancreatic, breast, and colorectal cancers, have revealed that CAFs can be classified into distinct subtypes with specialized functions, such as extracellular matrix (ECM) remodeling, immunomodulation, and metabolic reprogramming (Zhang et al., 2023; Pei et al., 2023). Beyond structural support, CAFs can enhance tumor proliferation and invasiveness via paracrine signaling. Importantly, several studies have shown that CAFs can reprogram cancer cell by promoting lipid and glucose metabolisms, either through direct metabolite exchange or paracrine signaling (Zhang et al., 2022; Niu et al., 2024; He et al., 2025). In CRC, CAFs have been implicated in promoting tumor growth, mediating resistance to therapy, and facilitating immune evasion through secretion of cytokines, growth factors, and matrix components. Importantly, CAFs engage in dynamic crosstalk with malignant cells, contributing to the establishment of a tumor-promotive niche (Kobayashi et al., 2022). Despite this, the precise spatial and molecular mechanisms underlying CAF-tumor interactions in metastatic CRC remain poorly understood, particularly within liver metastatic lesions where the stromal landscape is uniquely reprogrammed.

To address these knowledge gaps, we constructed an integrated single-cell and spatial atlas of CRC ecosystems, analyzing 35 publicly available scRNA-seq datasets encompassing normal colorectal tissue, normal liver, primary CRC tumors, and matched liver metastases. Using a comprehensive analytic pipeline, we identified a transcriptionally and metabolically distinct epithelial subpopulation, termed High-Malignancy CRC (High-M CRC), that is enriched in liver metastases and characterized by enhanced stemness and elevated glycolytic activity. Notably, this subpopulation exhibited myelocytomatosis oncogene (MYC)-driven transcriptional programming and engaged in spatially restricted interactions with certain subtypes of CAFs via the HGF-MET signaling axis. Spatial transcriptomics further validated the co-localization of these vital cells or factors, revealing a tightly organized metastatic ecosystem.

The structure of this study reflects the stepwise logic of our investigation, beginning with a pan-tissue comparisons. We focus on the malignant hierarchy within the epithelial compartment, followed by investigation into the stromal-tumor communication. Finally, we integrate ST data to illustrate these interactions within the physical landscape of the metastatic niche. This multi-layered research not only strengthens our understanding of the spatial and cellular dynamics of CRC progression, but also provides a conceptual framework for identifying intervention points within cancer metastasis.

## Materials and methods

### Datasets acquisition and data preprocessing

A total of 35 single-cell RNA-seq (scRNA-seq) datasets and two spatial transcriptomics datasets of CRC were retrieved from the GEO database (accessions GSE231559: samples GSM7290760-GSM7290785, GSE234804: samples GSM7474991-GSM7474999; GSE226997: sample P1 and P4). The scRNA-seq datasets encompasses CRC primary tumors (n = 9), matched liver metastases (n = 15), normal colorectal tissues (n = 3), and normal liver tissues (n = 8). Bulk RNA-seq data of CRC was collected from COAD (colon adenocarcinoma) dataset (n = 471) form The Cancer Genome Atlas (TCGA) database. We first performed stringent quality control on each independent sequencing library. Low-quality cells were filtered out based on the following criteria: high mitochondrial gene content (>25%) and fewer than 3 genes detected. Furthermore, only genes detected in at least 200 cells were retained. We employed the Seurat workflow for standardizing the raw count data. The ‘NormalizeData’ function was used to perform library size normalization for each cell, followed by log-transformation. The ‘FindVariableFeatures’ function was used to identify the top 2000 highly variable genes. Gene expression values were then scaled using the ‘ScaleData’ function, during which we regressed out the variable of mitochondrial gene percentage. To remove technically driven batch effects, we integrated the data using the widely recognized Harmony algorithm. The effectiveness of batch correction was visually confirmed by UMAP plots colored by sample origin, demonstrating effective mixing of cells from different cohorts while preserving biologically distinct clusters (Supplementary Figure S1B). Using the R package “Seurat” (v4.0) in the scRNA-seq raw data processing, we performed unsupervised clustering at resolution = 0.2 and annotated cell types based on canonical lineage markers. Data normalization was performed using LogNormalize function with a scale factor of 10,000. Gene expression values were log2-transformed and normalized using Transcripts Per Million (TPM). Cell clusters were identified via Seurat’s FindClusters function, and cell types (e.g., fibroblast, macrophage, T cell) were annotated using well-defined marker genes. Differentially expressed genes (DEGs) were determined at the cut-off values of pct = 0.25 and logFC = 0.25. R package “CellChat” (v1.6.0) was applied to dissect interaction networks between different cell types. Interaction weights and pathway strengths were calculated using default parameters.

### Malignant epithelial stratification and fibroblast subclustering

Epithelial cells were isolated and subclustered at resolution = 0.2 to identify 9 transcriptional states. Malignancy stratification integrated inferCNV chromosomal expression aberrations and tissue distributions. InferCNV (infer copy number variation) is employed to explore tumor scRNA-seq datasets to detect evidence of large-scale somatic chromosomal copy number alterations, such as gains or losses of entire chromosomes or large chromosomal segments. Specifically, clusters with inferCNV >3,300 classified as High-M CRC, inferCNV 2,300 to 3,300 as Low-M CRC, and inferCNV <2,300 as normal epithelium. The R package “CytoTRACE2” was used to quantify stemness potentials, while pseudotemporal trajectories were reconstructed using R package “Monocle2” based on top 2,000 variable genes. Distribution validation included Fisher’s exact test significance, along with OR (odds ratio) and Ro/e (observed/expected) ratios by calTissueDist function.

Fibroblast heterogeneity was resolved through subclustering at resolution = 0.2 based on marker expression and tissue distribution, characterizing their protumorigenic metabolic reprogramming and pathway activities to establish the mechanistic foundation for stromal-tumor crosstalk in metastatic CRC. Six CAF subtypes (mCAFs, myCAFs, iCAFs, neuro-like CAFs, EMT-like CAFs, and NFs) were defined based on consensus marker and literature-derived profiles (Lavie et al., 2022; Tsoumakidou, 2023). The lists of top expressed genes can be referred in Supplementary Material.

### Functional analysis and metabolic profiling

Gene functional and metabolic analyses were used to estimate the stemness properties, oncogenic pathway enrichment, and glycolytic dependencies in epithelial subpopulations and fibroblast subtypes. Transcriptional signatures underwent multi-method interrogation, including HALLMARK pathway enrichment used four irGSEA algorithms (AUCell, UCell, singscore, and ssGSEA). Gene Ontology-biological process (GO-BP) terms were analyzed via R package “clusterProfiler” (FDR <0.05). Metabolic flux quantification applied R package “scMetabolism” to KEGG and Reactome pathways, computing glycolysis and OXPHOS activity scores as gene set z-scores. Differential gene expression was determined using Seurat’s FindMarkers (Wilcoxon test, thresholds logFC = 0.25, pct = 0.25, FDR <0.05).

### Transcriptional regulator prediction

Transcriptional factor (TF) activity screening in High-M CRC was performed using dual computational frameworks. The DoRothEA algorithm employed regulon-based TF activity inference via VIPER scoring with normalized enrichment score (NES) thresholds >1.5 and permutation p-value <0.01. The Metascape tool leveraged integrated TF-target databases (e.g., TRRUST, ENCODE) to prioritize regulators using hypergeometric enrichment tests (FDR <0.05). TFs were ranked by combinatorial significance across both platforms, with spatial validation of MYC expression patterns through density UMAP.

### Bulk transcriptomic correlation analysis

Bulk transcriptomic correlation analysis was performed to validate scRNA-seq-derived ligand-receptor interactions using TCGA-COAD cohort data. Raw FPKM values were converted to TPM, log2 (TPM+1) transformed. Pairwise Pearson correlations between target gene pairs (e.g., MET-MYC, HGF-MYC) were computed using the ENCORI platform with strict filtering.

### Cell-cell and ligand-receptor interaction analysis

Cellular crosstalk analysis was performed to quantitatively resolve ligand-receptor interactions between CAF subtypes and epithelial subpopulations using an integrated framework combining R package “CellChat” and “NicheNet”. Communication probabilities and pathway flux quantifications established dominant senders and receivers. NicheNet prioritized key ligands using the fibroblast subtype-specific genes (logFC >1.5 vs. other fibroblasts; p < 0.05) against High-M CRC-specific receptors (logFC >1.5 vs. normal; pct ≥ 0.1; p < 0.05), limiting to genes expressed in more than 10% target cells and requiring Pearson correlation >0.85 for target predictions.

### Spatial expression and distribution analysis

Each spatial transcriptomics sample was independently normalized using the SCTransform method, which corrects for library size differences and technical covariates via regularized negative binomial regression. As the two spatial samples were analyzed separately, no cross-sample integration was performed. Spatial validation framework was performed to spatially resolve HGF-MET-MYC-glycolysis interactions through independent analysis of two CRC transcriptomics datasets (GEO: GSE226997 P1/P4) using AddModuleScore function for region-specific cell state mapping (top 100 logFC-ranked markers for mCAFs and High-M CRC), metabolic activity quantification, and ligand-receptor co-localization, thereby confirming juxtaposed niches of mCAFs and High-M CRC exhibiting spatially coupled HGF-MET signaling, MYC activation, and glycolytic hotspots in metastatic lesions.

### Cell culture

The cell lines utilized in this research were obtained from the American Type Culture Collection (ATCC, Manassas, United States). HCT-116 and COLO205 cells were maintained in Minimum Essential Medium (MEM), with each medium containing 10% fetal bovine serum (FBS) (Gibco, Shanghai, China). These cell lines were incubated at 37 °C in a 5% CO_2_ atmosphere within a humidified chamber.

### Immunohistochemistry (IHC) analysis

Immunohistochemistry (IHC) was performed to detect target proteins in primary CRC tissue samples. The protocol proceeded as follows: Tissue sections were first incubated at 55–60 °C for 2 h, followed by deparaffinization in xylene and gradual rehydration through an alcohol gradient (95%, 85%, and 75%).

For antigen retrieval, slides were immersed in EDTA buffer and subjected to microwave treatment - 6 min at medium-high power followed by 15 min at medium-low power. To block endogenous peroxidase activity, samples were treated with 3% hydrogen peroxide for 15 min, then incubated with goat serum for 30 min at room temperature (25 °C).

Primary antibody incubation was carried out at 4 °C overnight, followed by secondary antibody application at room temperature for 30–60 min. Finally, sections were developed using 3,3′-Diaminobenzidine (DAB), counterstained with hematoxylin, and imaged using a Nikon Eclipse 80i microscope (Nikon, Tokyo, Japan).

### Enzyme-linked immunosorbent assay (ELISA)

The Human HGF ELISA Kit protocol was followed to quantify HGF levels. Briefly, culture media samples from CRC cells were added to the assay plates, followed by incubation with HGF antibody at room temperature for 1 h. After six PBST washes, TMB Development Solution was added for 15 min before terminating the reaction with 100 µL of Stop Solution. Optical density readings were taken at 450 nm and 570 nm.

### Lentivirus infection and cell transfection

HGF sequence was cloned into a lentiviral vector and transduced into HEK293T cells for viral amplification. The resulting lentiviral particles were then purified and used to infect CAFs, establishing stable overexpression cell lines. Successful transduction was achieved through puromycin selection over a 14-day period.

### Transwell assays

Cell suspensions were prepared in serum-free medium. The lower chamber of a 24-well Transwell plate (8 μm pore size; Corning, United States) was filled with 600 μL of medium containing 5% FBS, while 300 μL of cell suspension (2 × 10^5^ cells) was added to the upper chamber. After 12 h of incubation at 37 °C in a humidified atmosphere, the cells were fixed with 4% paraformaldehyde for 30 min and stained with crystal violet for 20 min. Invading cells were quantified by counting three random fields per well using ImageJ software based on images captured with an inverted microscope.

### CCK-8 cell viability assay

Cells were plated in 96-well plates at a density of 2,000 cells/well in 100 μL of complete medium. After 24 h of incubation, the medium was replaced with 100 μL of fresh complete medium containing 10 μL CCK-8 reagent. Following incubation, absorbance readings at 450 nm were obtained using a spectrophotometer (Thermo Scientific, Pittsburgh, PA, United States) to assess cell viability.

### Statistical analysis

All statistical analyses were conducted in R v4.1.0, with package dependency management handled via renv (v0.15.5). Continuous variable normality was assessed using the Shapiro-Wilk test. Normally distributed data were compared via independent t-tests; non-normal distributions underwent analysis with the Mann-Whitney U test. Categorical variables were evaluated using chi-square tests supplemented by Fisher’s exact tests for sparse contingency tables. Statistical significance was defined as p < 0.05 (two-tailed).

## Results

### Single-cell atlas of CRC ecosystems reveals metastatic niche composition

**FIGURE 1:**
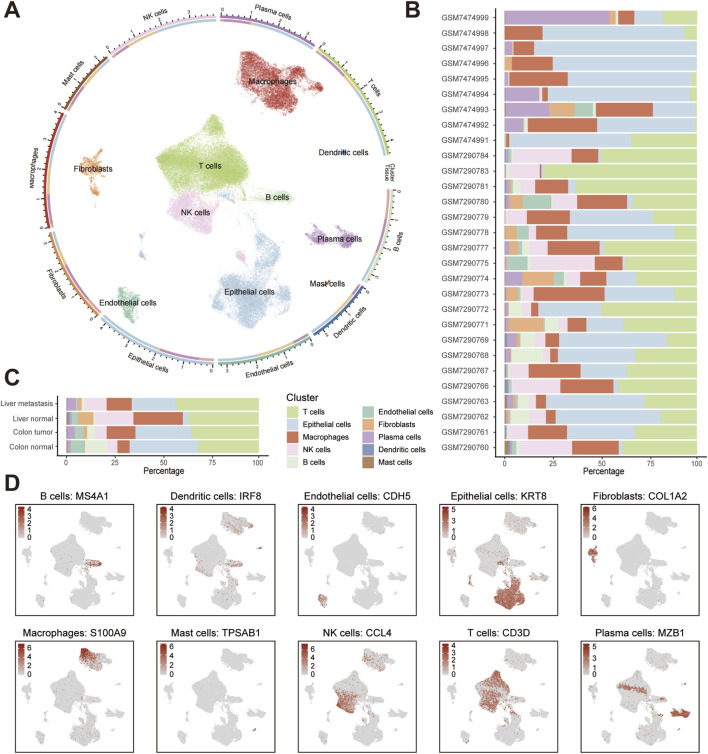
Single-cell landscape of colorectal cancer primary tumors, liver metastases, and normal tissues. **(A)** UMAP visualization colored by annotated cell types. Cell density-based contours have been removed for clarity, with distinct colors representing different cell types. **(B)** Stacked barplot showing cell-type proportions across scRNA-seq datasets. **(C)** Stacked barplot showing cell-type proportions across tissue types. **(D)** UMAP feature plots of cell types with canonical marker.

We integrated and analyzed 35 scRNA-seq datasets from the GEO database, encompassing colorectal cancer primary tumors, matched liver metastases, normal colorectal mucosa, and normal liver tissues. As the results demonstrate, the circled UMAP visualization resolved 10 transcriptionally distinct cell populations across all samples (Figure 1A). Analysis of cellular fractions across datasets demonstrated significant heterogeneity in the proportions of especially epithelial cells and major immune cell types (e.g., T cells, NK cells, and macrophages) (Figure 1B). When aggregated by tissue origin, T cell infiltration dominated liver metastases, whereas normal colon tissues exhibited minimal T cell presence. This distribution inversely correlated with epithelial abundance, which peaked in normal colon and reached minimal levels in liver metastases (Figure 1C). Original UMAPs of whole cells and tissue-specific cells are shown in Supplementary Figure S1. We also provide a UMAP visualization colored by sample origin to demonstrate successful batch effect correction after Harmony integration (Supplementary Figure S1B). Marker expression validation confirmed annotation robustness, with one single identifier for each cell type (e.g., KRT8 for epithelium, COL1A2 for fibroblasts) exhibiting compartment-specific localization in UMAP space (Figure 1D). More established markers for cells are shown in Supplementary Figure S1. The systematic remodeling of cellular architecture in metastatic sites, characterized by immune cells accumulation, establishes liver metastases as immunologically privileged niches warranting mechanistic investigation.

### Epithelial subclustering identifies highly malignant subpopulations in CRC metastasis

**FIGURE 2:**
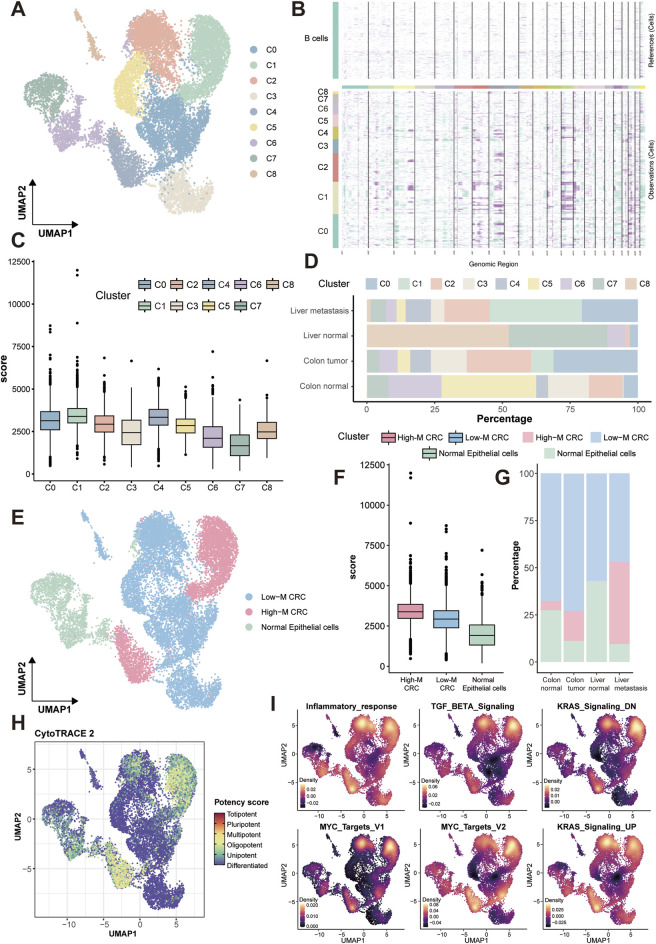
Malignant epithelial stratification, distribution and functional enrichment. **(A)** UMAP of nine transcriptionally distinct epithelial subclusters, annotated as: stress-responsive tumor cells (C0), cancer stem cell-like population (C1), metabolically active tumor cells (C2), goblet cell-like differentiated tumor cells (C3), G2/M-phase enriched highly proliferative tumor cells (C4), and colonocyte-like differentiated tumor cells (C8). **(B)** Heatmap of chromosomal relative expression in each subcluster via inferCNV analysis. Each row stands for a cell, and each column stands for a gene. Darker the bar is, more CNVs the cells have. **(C)** Box plot of inferCNV scores of each subcluster. **(D)** Tissue-specific distribution of subclusters. **(E)** UMAP of three re-annotated epithelial subpopulations. **(F)** Box plot of inferCNV scores of each subpopulation. **(G)** Tissue-specific distribution of subpopulations. **(H)** UMAP of stemness scores of subpopulations. **(I)** Density plots of six malignancy-associated hallmark pathways of subpopulations. CNVs, copy number alterations.

As the epithelium is the origin and main components of colorectal tumor, we subclustered epithelial cells (resolution = 0.2) into 9 transcriptionally distinct clusters (Figure 2A) and integrated inferCNV scores to stratify cell populations based on their malignancy (Figures 2B,C). Through systematic annotation based on canonical marker genes, we identified these clusters as: stress-responsive tumor cells (Cluster 0, marked by HIST1H2BG, JUN, EGR1, ATF3), cancer stem cell-like population (Cluster 1, LGR5, PROM1), metabolically active tumor cells (Cluster 2, EIF/EEF genes, CCND1), goblet cell-like differentiated tumor cells (Cluster 3, MUC2, TFF3, SPDEF), G2/M-phase enriched highly proliferative tumor cells (Cluster 4, CDK1, CCNB1/2, PLK1, AURKA/B, TOP2A, MKI67), and colonocyte-like differentiated tumor cells (Cluster 8, KRT20, CEACAM7, FABP1/2, SLC26A3, HMGCS2). Notably, the cancer stem cell-like (Cluster 1) and highly proliferative (Cluster 4) subpopulations constituted the core components of the High-M CRC subtype identified through inferCNV scoring (Figure 2D). Based on inferCNV scores and tissue distribution patterns, we stratified the epithelial cells into high-malignancy CRC (High-M CRC), low-malignancy CRC (Low-M CRC), and normal epithelial cells (Figures 2E,F). The complete lists of differentially expressed genes for all 9 epithelial subclusters are provided in the Supplementary Table S1, facilitating in-depth exploration of their transcriptional profiles. The distribution patterns of these three subpopulations across tissues confirmed the validity of re-annotation and classification, demonstrating that High-M exhibits the highest prevalence in liver metastatic lesions, whereas normal epithelial cells display a greater proportion in normal tissues compared to the other two tumor types (Figure 2G). We note that the UMAP topology of epithelial cells in Figure 2 differs from that in Figure 1A, as it results from a dedicated re-analysis of the epithelial subset, which more clearly reveals intra-epithelial heterogeneity. Further calTissueDist analysis calculated the OR (odds ratio) value and Ro/e (ratio of observed over expected cell numbers) value for each subpopulation in different tissue types, and liver metastasis obtained the highest values of OR and Ro/e in High-M CRC subpopulation (Supplementary Figure S2).

Subsequently, tumor stemness was assessed by Potency score using CytoTRACE2 analysis, which revealed significantly enhanced stemness characteristics in the High-M CRC subpopulation (Figure 2H). Further HALLMARK pathway analysis demonstrated markedly and specific enrichment of six representative malignancy-associated pathways in this subpopulation, including inflammatory response, TGF-β signaling, KRAS-dysregulated genes (down-regulated and up-regulated) signaling, and Myc targets (Figure 2I). Subsequently, to further elucidate pathway alterations in cellular subpopulations, we performed another HALLMARK pathway enrichment analysis using the irGSEA package. Results from four distinct computational methods, AUCell, UCell, singscore, and ssgsea, were comparatively presented (Supplementary Figure S3). This analysis substantiated elevated enrichment levels of malignancy-associated pathways in High-M CRC; as well as revealing overlapping upregulation of the “Glycolysis” signaling pathway.

### Glycolytic reprogramming drives malignant progression in metastatic CRC

**FIGURE 3:**
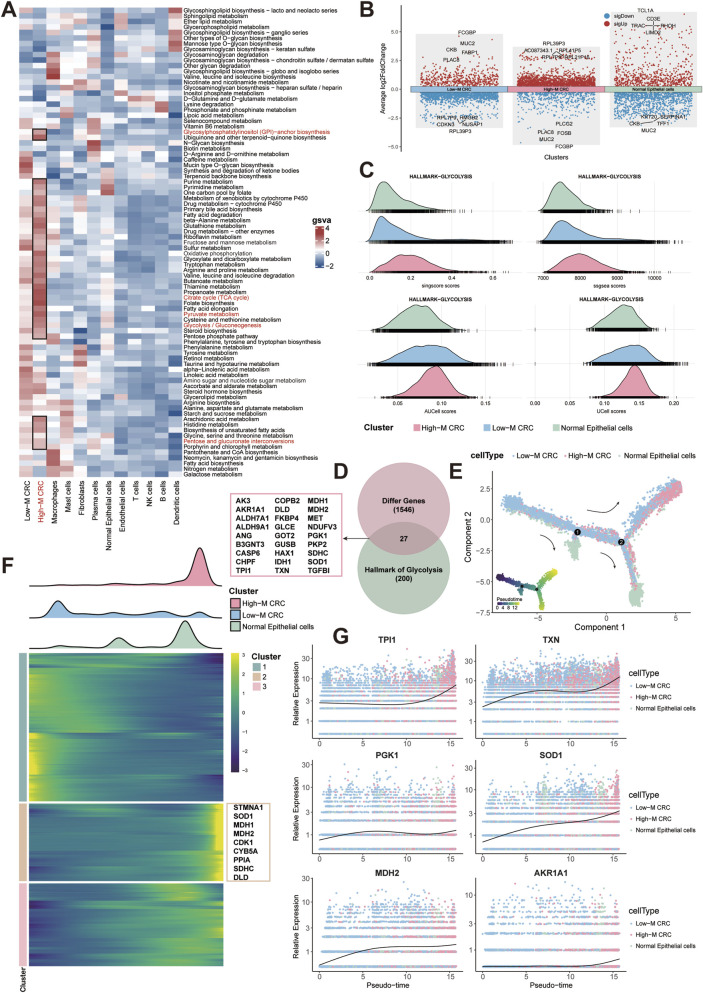
Glycolytic remodeling in CRC malignant progression. **(A)** Metabolic activity by epithelial and other cell type. **(B)** Dot plots of DEGs between three subpopulations. **(C)** Ridgeline plots of glycolytic activity across three subpopulations using four algorithms. **(D)** Venn diagram of overlapped hub glycolytic genes. **(E)** Trajectory analysis of three subpopulations. **(F)** Pseudotemporal heatmap of top 2000 genes in three subpopulations. **(G)** Expression of hub glycolytic gene over pseudotime.

Based on the results of irGSEA analysis,we further delineated the integrated metabolic profiling of epithelial cells and other cell types using “scMetabolism” algorithm, uncovering the glycolytic dependency in HM-CRC (Figure 3A). The dot plots showed the DEGs of three subpopulations and the top-five genes were noted (pct = 0.25, logFC = 0.25, p = 0.01, Figure 3B). The ridgeline plot demonstrates findings consistent with prior analytical outcomes by four independent algorithms, revealing significantly enhanced glycolytic activity in High-M CRC group (Figure 3C).

We next intersected the upregulated genes in High-M CRC with the glycolysis gene set from the HALLMARK collection, and finally identified 27 hub genes (Figure 3D). Pseudotime trajectory analysis delineating developmental pathways among the three epithelial subpopulations revealed that High-M CRC originates from Low-M CRC differentiation (Figure 3E). Visualization of the top 2,000 differentiation-associated genes in a pseudotime heatmap implicated key glycolytic enzymes in promoting malignant progression of CRC (e.g., STMNA1, SOD1, MDH1/2) (Figure 3F). Finally, expression profiles of the 27 hub genes across epithelial subpopulations were plotted along the pseudotemporal continuum, demonstrating elevated expression of critical genes (e.g., TPI1, TXN) in High-M CRC subpopulation over developmental time (Figure 3G; Supplementary Figure S4). This pseudotemporal metabolic escalation defines a targetable axis in metastatic evolution.

### MYC transcriptionally regulates glycolysis in High-M CRC and interacts with fibroblast

**FIGURE 4:**
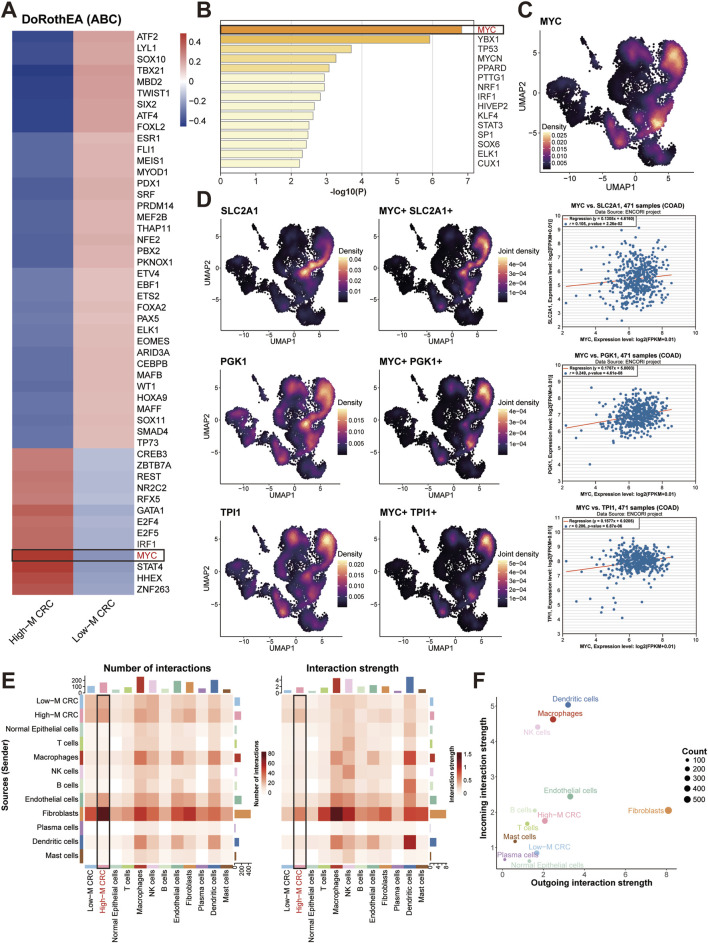
MYC regulation on glycolysis and stromal crosstalk. **(A,B)** Heatmaps of TF prioritization generated by DoRothEA algorithm **(A)** or Metascape toole **(B)**. **(C)** MYC expression density of three subpopulations. **(D)** MYC-glycolytic gene co-expression and expression correlation. **(E)** Cell-cell interaction weights between CRC cells and other cells. **(F)** Dot plot of income and outgoing interaction strengths of cellchat analysis.

To discover the key molecule and regulatory mechanisms in High-M CRC, we performed transcriptional regulator screening and identified MYC as the top-ranked transcription factor (TF) in HM-CRC (Figures 4A,B). Density plots depict expression profiles and spatial distribution of MYC within CRC tissues (Figure 4C). Subsequent single-cell analysis mapped expression patterns of three representative glycolysis-associated genes (SLC2A1, PGK1, TPI1), with co-localization analysis revealing significantly enriched co-expression density within the High-M CRC subpopulation (Figure 4D). Correlation analysis was then performed using bulk RNA-seq data from TCGA-COAD samples; employing the ENCORI platform, we generated positively expressive correlation scatter plots between MYC and these pivotal genes.

Finally, CellChat-mediated cellular communication analysis identified prominent ligand-receptor interaction intensity and quantity between cancer-associated fibroblasts and High-M CRC cells (Figures 4E,F). Collectively, these findings underscore the regulatory role of the TF MYC in modulating glycolytic metabolism in High-M CRC, and highlighted the research significance of cancer-associated fibroblasts (CAFs) as potential interaction partners for tumor cell.

### CAFs subclustering identifies malignancy-associated distribution, metabolism, and functional enrichment

**FIGURE 5:**
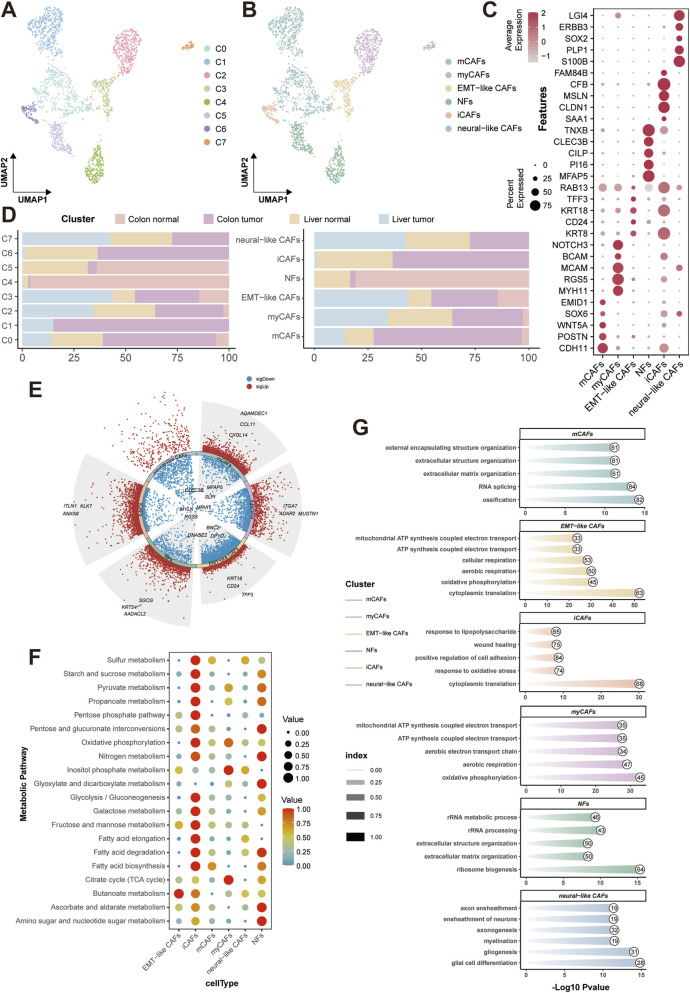
CRC-associated fibroblast subtypes characterization and functional analysis. **(A)** UMAP of fibroblast subclustering. **(B)** UMAP of fibroblast functional annotation. **(C)** Dot plot of CAFs marker. **(D)** Tissue distribution of different fibroblast subclusters and subtypes. **(E)** DEG volcano plot of different CAFs. **(F)** Metabolic profiling of different CAFs. **(G)** GO-BP enrichment of CAFs. GO-BP, Gene Ontology-biological process.

Given the significance of CAFs-tumor crosstalk identified in our prior analysis, we interrogated the fibroblast heterogeneity by subclustering all fibroblasts (resolution = 0.1), resolving eight distinct subclusters (C0 to C7) (Figure 5A). Subsequent annotation leveraging tissue-specific markers delineated six functional subtypes: matrix CAFs (mCAFs), myofibroblasts (myCAFs), inflammatory CAFs (iCAF), EMT-like CAFs, neural-like CAFs, and normal fibroblasts (NFs) (Figure 5B). Dot plot visualization confirmed subtype-specific enrichment of canonical markers (Figure 5C). Subsequent distribution analysis revealed profound tissue-specific compartmentalization. Metastasis tissue exhibited dominance of protumorigenic CAFs, such as EMT-like CAFs, myCAFs, and neural-like CAFs. Colon tumor showed enrichment of iCAFs and mCAFs (Figure 5D). Volcano plots of DEGs (pct = 0.25, logFC = 0.25, p = 0.05) highlighted the top upregulated genes in CAFs subtypes (Figure 5E).

To explore the associated role of CAFs in metabolism, metabolic profiling via scMetabolism was depicted and demonstrated pronounced metabolic activation in iCAFs, with especially elevated glycolysis and gluconeogenesis (Figure 5F). Meantime, GO-BP enrichment further exposed functional specialization of different subtypes (Figure 5G). The clustering and functional analyses on CAFs reflect their protumorigenic role in the TME of CRC. Hence, the specific interaction between CAFs and CRC malignancy urgently needs further investigation in the following research.

### CAFs-tumor crosstalk reveals activated HGF-MET-MYC signaling axis in High-M CRC

**FIGURE 6:**
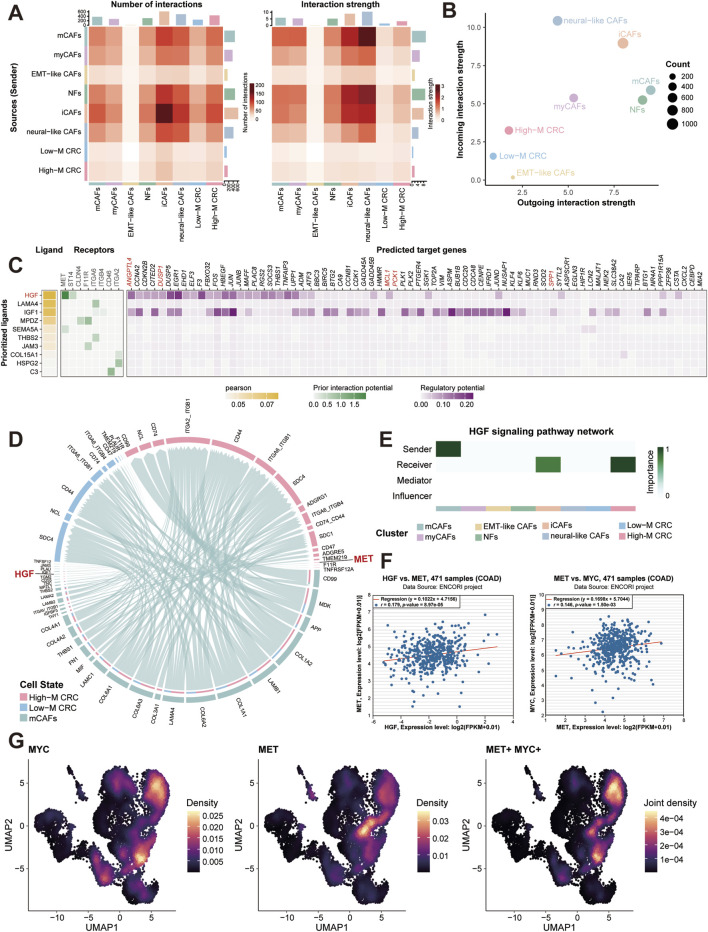
Stromal-tumor crosstalk drives MYC activation via HGF-MET signaling. **(A)** CellChat interaction weights between fibroblast subtypes and epithelial subpopulations. **(B)** Outgoing and incoming signal strengths across major cell types. **(C)** Heatmap of ligand-receptor analysis between CAFs and High-M CRC cells. **(D)** HGF-MET interaction network between CAFs and CRC cells. **(E)** HGF signaling pathway network analysis. **(F)** Correlation analysis of MET and MYC expression in TCGA-COAD cohort. **(G)** Single-cell co-expression density of MET and MYC in High-M CRC. COAD, colon adenocarcinoma; CRC, colorectal cancer; HGF, hepatocyte growth factorl; MET, mesenchymal to epithelial transition factor; MYC, myelocytomatosis oncogene.

Correspondingly, we dissected stromal-epithelial communication building on fibroblast heterogeneity. CellChat analysis quantified interaction weights between fibroblast subtypes and epithelial subpopulations, revealing iCAFs and mCAFs as dominant communicators with High-M CRC cells (Figure 6A). The global signaling patterns further identified them as the top signal senders and receivers, respectively (Figure 6B).

To pinpoint key molecular interaction, we employed NicheNet tool to conduct ligand-receptor analysis. As the results showed, this prioritized hepatocyte growth factor (HGF) as the top ligand, with its receptor mesenchymal to epithelial transition factor (MET) highly expressed in High-M CRC (Figure 6C). The predicted targets of the HGF-MET signaling showed several glucose metabolism-associated genes, such as ANGPTL4, DUSP1, PCK1, and SPP1, which participated in the regulation of glycolysis and gluconeogenesis. CellChat analysis validation confirmed HGF-MET interactions exclusively enriched in mCAFs and High-M CRC pairs (Figure 6D; Supplementary Figure S5), consistent with literature implicating HGF-MET signaling in MYC induction (Chu et al., 2022; Li et al., 2008). Meantime, the HGF signaling pathway network analysis was conducted between different cell types. The result further confirmed the importance of High-M CRC as the receiver of HGF which potentially induced the MYC upregulation (Figure 6E).

Similarly, the correlation analysis was then performed using bulk RNA-seq data from TCGA-COAD samples. The positively correlated relationships between MET and HGF/MYC expression were generated (Figure 6F). Besides, the single-cell spatial density mapping revealed MET-MYC co-localization within High-M CRC niches (Figure 6G). These findings establish mCAF-derived HGF as a key regulator of MET-MYC signaling in metastatic CRC.

### Spatial mapping validates elevated HGF-MET-MYC-glycolysis niches in High-M CRC

**FIGURE 7:**
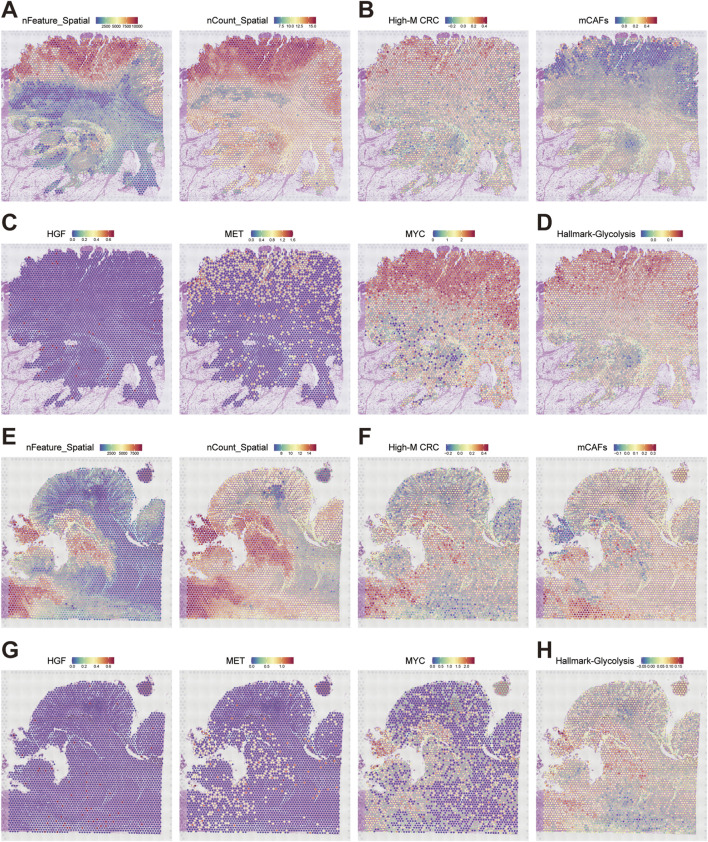
Spatial architecture of HGF signaling-mediated glycolytic niches in CRC metastasis. **(A,E)** Quality metrics nFeature and log (nCount) of ST dataset GSE226997 P1 and P4. **(B,F)** Spatial mapping of High-M CRC cell and mCAFs probabilities in two datasets. **(C,G)** Spatial expression maps of HGF, MET, and MYC in two datasets. **(D,H)** Predicted spatial activity maps of glycolysis in two datasets.

To spatially resolve the HGF-MET-MYC signaling-medicated glycolysis axis, we obtained and analyzed two CRC ST datasets from GEO database (GSE226997, samples P1/P4). Quality control confirmed high transcript coverage (Figures 7A,E). Using fibroblast subtype markers from scRNA-seq (logFC-ranked top 100 for mCAFs and High-M CRC, Supplementary Material), we applied AddModuleScore tool to infer spatial distributions. This revealed juxtaposed niches of High-M CRC subpopulation and mCAFs in the ST sample with direct physical adjacency (Figures 7B,F). Based on the transcriptomics data, we generated the spatial expression mapping of HGF (stroma-enriched), MET, and MYC (tumor-enriched), and confirmed their co-localization within High-M CRC regions bordering mCAFs (Figures 7C,G).

Finally, the glycolytic activity was spatially resolved according to glycolysis Hallmark genes as well. The results demonstrated precise overlap of elevated glycolysis, MYC expression, and MET hotspots within High-M CRC regions adjacent to mCAFs (Figures 7D,H). These spatially resolved ecosystems revealed highly activated HGF-MET-MYC-glycolysis signaling in High-M CRC, and define is as a fundamental unit of CRC metastasis.

### CAFs activate the malignant phenotype of CRC through the HGF/MET/MYC signaling axis

**FIGURE 8:**
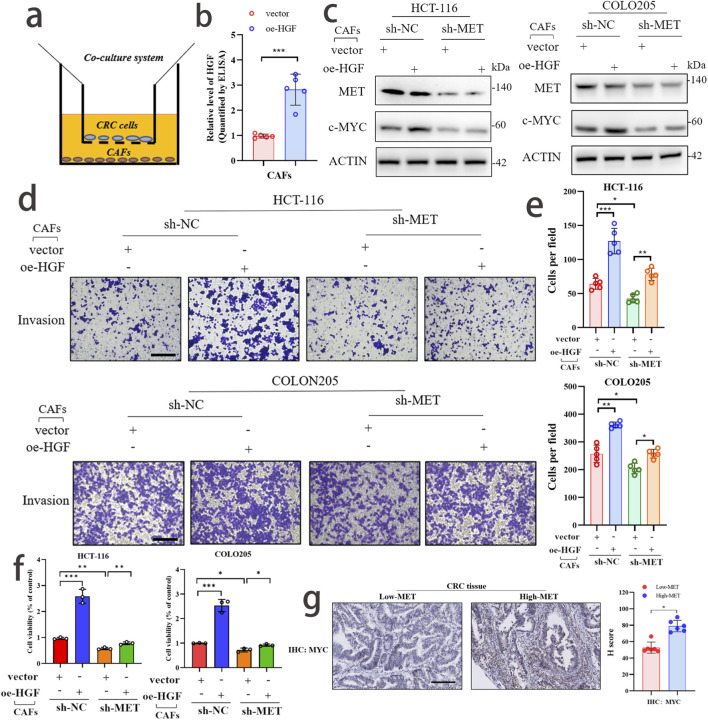
CAFs activate the malignant phenotype of CRC through the HGF/MET/MYC signaling axis. **(a)** Schematic diagram of co-culture between CAF and CRC. **(b)** The efficiency of HGF overexpression in CAF cells was detected by ELISA assay. **(c)** WB analysis confirmed that knockdown of MET expression could reverse the promoting effect of HGF overexpression on MYC expression. **(d,e)** Transwell assay demonstrated that knockdown of MET expression could reverse the promoting effect of HGF overexpression on the invasive phenotype of CRC. scale bar: 50 μm **(f)** CCK-8 assay confirmed that knockdown of MET expression could reverse the promoting effect of HGF overexpression on the proliferative phenotype of CRC. **(g)** The expression levels of MET and MYC in CRC samples and their correlation were detected by immunohistochemical experiments.

To explore the potential signaling axis (HGF/MET/MYC) between CAFs and CRC, we conducted further validation using colon cancer cell lines HCT-116 and COLO205. By isolating primary CAF cells, we co-cultured them with colon cancer cell lines *in vitro* (Figure 8a). Correspondingly, CAFs capable of stable passage were further treated with HGF overexpression (Figure 8b). At the protein level, we observed that knockdown of MET expression significantly reversed the upregulation of MYC expression in CRC cells co-cultured with HGF-overexpressing CAFs (Figure 8c). Through Transwell assay, we found that knockdown of MET expression significantly reversed the invasive ability of CRC cells co-cultured with HGF-overexpressing CAFs (Figures 8d,e). Additionally, in the CCK-8 assay, we also observed that knockdown of MET expression significantly reversed the proliferative trend of CRC cells co-cultured with HGF-overexpressing CAFs (Figure 8f). The above experimental results suggest that the HGF/MET/MYC signaling axis plays a crucial role in the interaction between CAFs and CRC. Finally, through immunohistochemical experiments, we observed that the expression level of MYC was higher in the MET high-expression group compared to the MET low-expression group. This suggests a positive correlation (Figure 8g).

## Discussion

Clarifying the cellular and molecular pathways underpinning CRC metastasis remains a major challenge in oncology. While prior studies have delineated several signaling cascades central to metastatic spread and TME remodeling (Li et al., 2024; Zhan et al., 2017; Liu et al., 2023), a spatially resolved single-cell atlas capturing the dynamic evolution of CRC across primary and metastatic sites has been lacking. To address this gap, we integrated 35 high-quality scRNA-seq datasets with spatial transcriptomics and pathway enrichment analyses, constructing a comprehensive landscape of cell-type-specific changes and intercellular communication in both primary CRC and liver metastases. Our approach revealed significant transcriptional heterogeneity across epithelial, stromal, and immune compartments, underscoring the architectural complexity of CRC progression. A key finding was the identification of a transcriptionally and metabolically distinct subpopulation of highly malignant CRC epithelial cells (termed High-M CRC), defined by elevated MYC-driven glycolytic activity and spatial coordination with metabolically active CAFs, particularly mCAFs, via the HGF-MET signaling axis.

Our integrated analysis, utilizing UMAP-based clustering, revealed robust inter- and intra-tumoral heterogeneity in both normal and malignant colorectal tissues, especially within epithelial and immune compartments (Chen et al., 2021). Notably, liver metastases exhibited significantly higher T cell infiltration compared to primary tumors and adjacent normal tissues, which inversely correlated with epithelial cell abundance. This suggests that epithelial attrition and immune cell recruitment may be tightly linked processes during metastatic colonization (Massagué and Ganesh, 2021). The elevated immune presence at metastatic sites aligns with the concept of metastases as “immune-modulated” or “immune-privileged” niches, a paradigm supported by findings in numerous cancers, where immune composition has been shown to regulate metastatic potential (Quah et al., 2023; Kim et al., 2021). The consistent marker expression and cluster-specific transcriptional profiles across datasets validate the robustness of our annotations, and underscore the reproducibility of cell state dynamics across diverse tissue contexts.

To dissect malignant heterogeneity within the epithelial compartment, we applied subclustering and inferCNV scoring, stratifying cells into normal, low-malignancy (Low-M), and high-malignancy (High-M) subtypes. High-M CRC cells were predominantly enriched in liver metastases, signifying both spatial and functional divergence from their normal and Low-M counterparts. These cells displayed elevated CytoTRACE scores, suggesting a proliferative, stem-like phenotype associated with aggressive oncogenic behavior (Gkountela and Aceto, 2016; Cañellas-Socias et al., 2022). For instance, Yao et al. found that SCF-FBXL8 axis contributes to liver metastasis and stem-cell-like features of CRC cells (Yao et al., 2023). Next, the Hallmark pathway enrichment across multiple scoring algorithms revealed consistent activation of malignancy-associated pathways such as TGF-β, KRAS, and MYC signaling (Hao et al., 2019; Guo et al., 2025; Meškytė et al., 2020). Strikingly, glycolytic reprogramming emerged as a defining feature of High-M cells, with bulk and single-cell metabolic profiling confirming marked upregulation of glycolysis-related genes. Differential expression analysis identified 27 glycolytic hub genes, including STMN1, SOD1, and TPI1, many of which are previously implicated in CRC metabolism and therapy resistance (Shi et al., 2020; Chen et al., 2022).

In cancer biology, glycolysis, the metabolic pathway that converts glucose into pyruvate, and the MYC oncogene are frequently reprogrammed to support the rapid proliferation and survival of cancer cells. This phenomenon, often termed the Warburg effect, describes the preferential reliance of cancer cells on aerobic glycolysis even in the presence of oxygen, distinguishing them from normal cells that primarily use oxidative phosphorylation for energy production (Koppenol et al., 2011). The precise mechanisms and clinical implications of this metabolic shift are critical areas of ongoing research in oncology. In CRC, the interplay between glycolysis and MYC-driven regulation is particularly significant for disease progression and metastasis. Recent studies highlight how highly malignant CRC epithelial cells, especially those found in metastatic sites, exhibit elevated MYC-driven glycolytic activity (Zhao et al., 2024). This metabolic adaptation not only provides the necessary energy and building blocks for rapid growth but also contributes to the aggressive, stem-like phenotype associated with advanced disease (Zhou et al., 2023; Liu et al., 2024).

Meantime, the pseudotime trajectory analysis delineated a continuous transition from Low-M to High-M states, characterized by progressive upregulation of glycolytic gene signatures. These findings suggest that metabolic adaptation not only accompanies but actively drives malignant progression in CRC. Transcription factor enrichment pinpointed MYC as the central regulator of this metabolic phenotype. Co-expression and spatial mapping further confirmed MYC’s tight spatial colocalization with key glycolysis genes. MYC, a powerful transcription factor, plays a central role in orchestrating this metabolic reprogramming by directly upregulating the expression of numerous glycolysis-related genes, such as SLC2A1 (encoding GLUT1, a glucose transporter) and HK2 (Zeng et al., 2024; Han et al., 2022). This direct transcriptional control by MYC ensures a sustained supply of glycolytic intermediates, fueling both energy production and biosynthetic pathways essential for tumor growth and invasion (Yeung et al., 2008). The following TCGA-based correlation analyses supported this relationship, reinforcing its dual role as both a downstream effector of oncogenic signaling and a vital driver of glycolytic reprogramming in CRC metastasis (Jing et al., 2022). The robust and recurrent MYC-glycolysis axis across spatial and single-cell modalities positions MYC as a promising therapeutic target, especially when considered in combination with glycolysis inhibitors or agents that disrupt stromal-epithelial crosstalk.

Beyond epithelial compartments, our study uncovered substantial heterogeneity among CAFs, revealing eight transcriptionally distinct subtypes. Pro-tumorigenic CAF subsets, particularly mCAFs and EMT-like CAFs, were enriched in metastatic samples, consistent with their proposed roles in fostering metastasis-supportive niches (Xu et al., 2022; Yao et al., 2024; Hu et al., 2019). Metabolic profiling indicated heightened glycolytic and gluconeogenic activity in iCAFs and mCAFs, suggesting a metabolically reprogrammed stromal environment that favors tumor progression. Using CellChat, we identified mCAFs as the dominant interactor with High-M epithelial cells and major producers of HGF, implicating them as key facilitators of the HGF-MET signaling axis. These findings reinforce mCAFs as regulators of spatially resolved metabolic and mitogenic signaling in metastatic tissues. Our integrative analysis highlights the HGF-MET-MYC pathway as a central mechanism underlying stromal-epithelial communication in CRC metastasis. The HGF-MET signaling has been extensively reported to participate in the tumorigenesis, metabolism and metastasis of various cancers, and multiple HGF-MET pathway inhibitors exhibited potent anti-cancer role by preventing tumor metastasis (Huang et al., 2019; Yin et al., 2019; Shi et al., 2021). Spatial transcriptomic validation confirmed this spatially structured interaction, with co-localization of mCAFs, HGF, MET, and MYC targets within specific tumor regions. These “metabolic hubs” appear to serve as focal points for tumor growth and niche remodeling. Similar spatially constrained interactions have been observed in hepatocellular carcinoma and lung adenocarcinoma, suggesting that such tumor-stromal metabolic units may represent a common mechanism across cancer types (Liu et al., 2025; Jain et al., 2023).

Despite the depth of our multi-omics integration, several limitations warrant consideration. First, our conclusions regarding malignancy trajectories and pathway activity are based on computational approaches, without direct experimental confirmation. Future studies incorporating lineage tracing or *in vivo* validation could provide stronger evidence. Second, while our dataset integration strategy accounted for batch effects and technical noise, the potential influence of patient heterogeneity, including treatment history and genetic background, remains a concern (Huang et al., 2023). Besides, while spatial transcriptomics enabled the mapping of cell-cell interactions, current technologies do not offer true single-cell resolution, potentially obscuring finer-scale spatial dynamics. Finally, although MYC and glycolysis represent potential therapeutic targets, direct inhibition of transcription factors like MYC remains a major pharmacological challenge. Indirect strategies, such as targeting upstream effectors (e.g., HGF) or modulating CAFs behaviors, may offer more feasible therapeutic avenues. Employing patient-derived organoids, xenografts, and CRISPR-based perturbation systems could validate these insights and identify actionable vulnerabilities in CRC metastasis (Michels et al., 2020).

## Conclusion

This study advances our understanding of mCRC by offering a high-resolution view of stromal-tumor interactions within the metastases. By integrating single-cell and spatial transcriptomic data, we uncover not only cellular diversity but also the spatial logic that governs malignant progression. The identification of MYC-driven metabolic reprogramming, mediated by fibroblast-derived signals, highlights the crucial role of the TME in shaping cancer cell behavior. These findings underscore the potential of targeting metabolic and stromal signaling pathways as therapeutic strategies. Looking forward, translating these insights into functional models and clinical contexts is promising to discover potential targets and guide precision oncology in CRC metastasis.

## Data Availability

The original contributions presented in the study are included in the article/Supplementary Material, further inquiries can be directed to the corresponding author.

## Supplementary material

SUPPLEMENTARY FIGURE S1 Identification of the single-cell constitution of the 35 scRNA-seq datasets. **(A)** UMAP depicting the cell subclustering. **(B)** UMAP visualization colored by sample origin, demonstrating effective batch effect correction after Harmony integration. **(C)** UMAP depicting the cell type distribution across tissues. **(D)** Established markers for 10 major cell types.

SUPPLEMENTARY FIGURE S2 Functional and developmental characterization of CRC subpopulations. **(A)** The OR (odds ratio) value and R_o/e_ (ratio of observed over expected cell numbers) value for each subpopulation in different tissue types. **(B)** The results of GO-BP analysis for each cell type. **(C,D)** The cancer stemness of different subpopulations by CytoTRACE2 analysis. **(E)** The developmental trajectory of subpopulations by Vector algorithm.

SUPPLEMENTARY FIGURE S3 Multi-algorithm Hallmark pathway enrichment in epithelial compartments. **(A-D)** This figure shows the HALLMARK pathway enrichment using R package “irGSEA” across normal epithelium, Low-M CRC, and High-M CRC using four independent algorithms.

SUPPLEMENTARY FIGURE S4 Pseudotemporal expression analysis of the rest 21 hub genes. This figure shows the pseudotime-dependent expression of the rest 21 hub genes across normal epithelium, Low-M CRC, and High-M CRC.

SUPPLEMENTARY FIGURE S5 Distribution of HGF-MET signaling in the stromal-tumor crosstalk of CRC. **(A)** Cellchat analysis of ligand-receptor communication between CAFs and CRC cells. **(B)** Density UMAP of HGF in fibroblasts. **(C-F)** Integrative cell-cell interaction analyses of HGF-MET signaling in CRC.

## Abbreviations

CAF, Cancer-associated fibroblasts; CRC, Colorectal cancer; CRLM, Colorectal liver metastasis; DEGs, Differentially expressed genes; HGF, Hepatocyte growth factor; High-M CRC, High-malignancy colorectal cancer (subpopulation); IHC, Immunohistochemistry; mCAFs, Matrix cancer-associated fibroblasts; MET, Mesenchymal-epithelial transition factor; MYC, MYC proto-oncogene, bHLH transcription factor; scRNA-seq, Single-cell RNA sequencing; ST, Spatial transcriptomics; TME, Tumor microenvironment; inferCNV, Inference of copy number variations; GO-BP, Gene Ontology-biological process.
