# Supplementary figures and images for: A spatially resolved single-cell landscape of colorectal cancer liver metastasis reveals a stromal-tumor glycolytic signaling interaction
Source: Front Cell Dev Biol. 2025 Oct 29;13:1687485. doi: 10.3389/fcell.2025.1687485 (PMC12605286; doi:10.3389/fcell.2025.1687485)

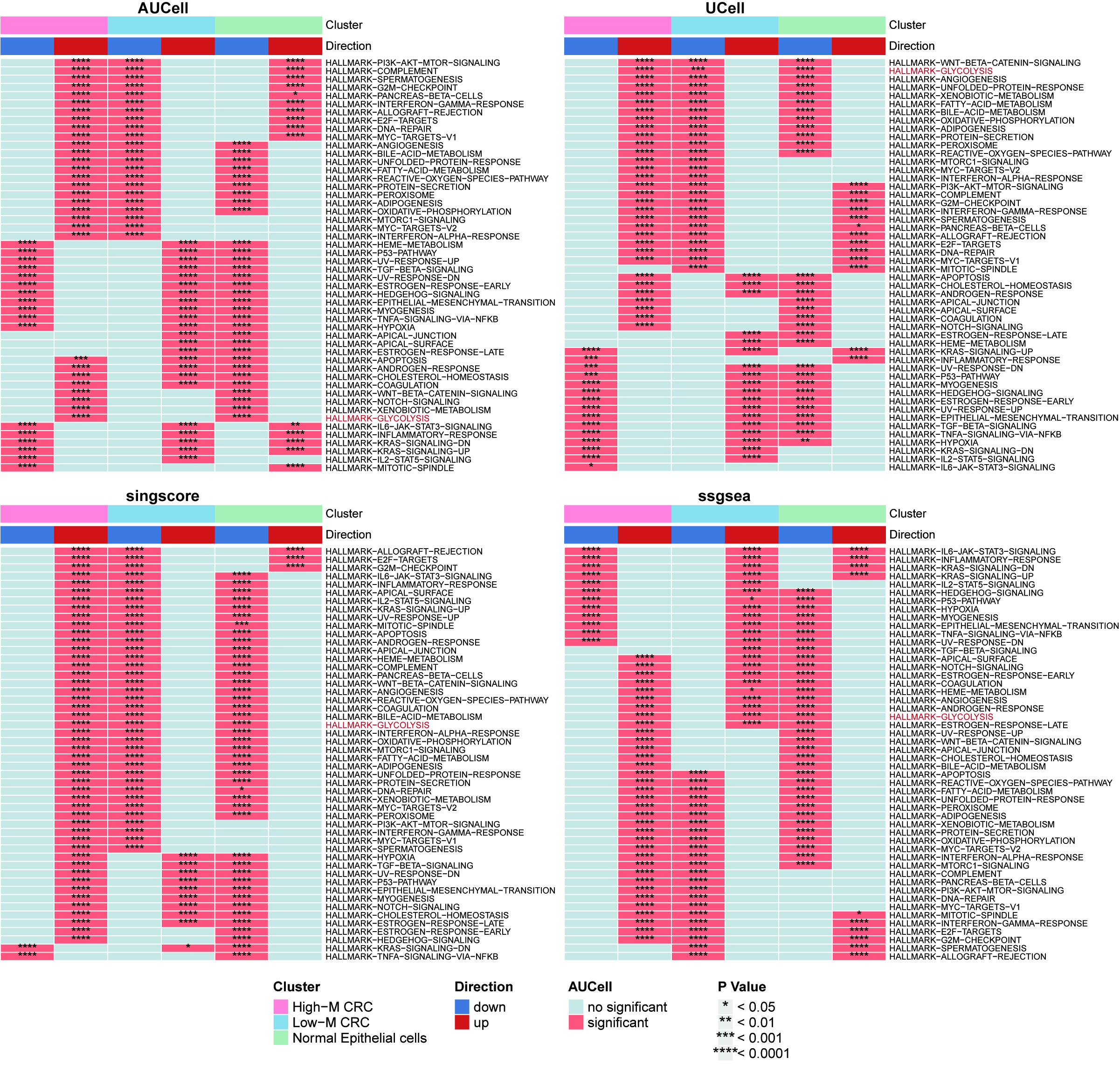

Supplement: Supplementary file 2 [file Image3.tif]

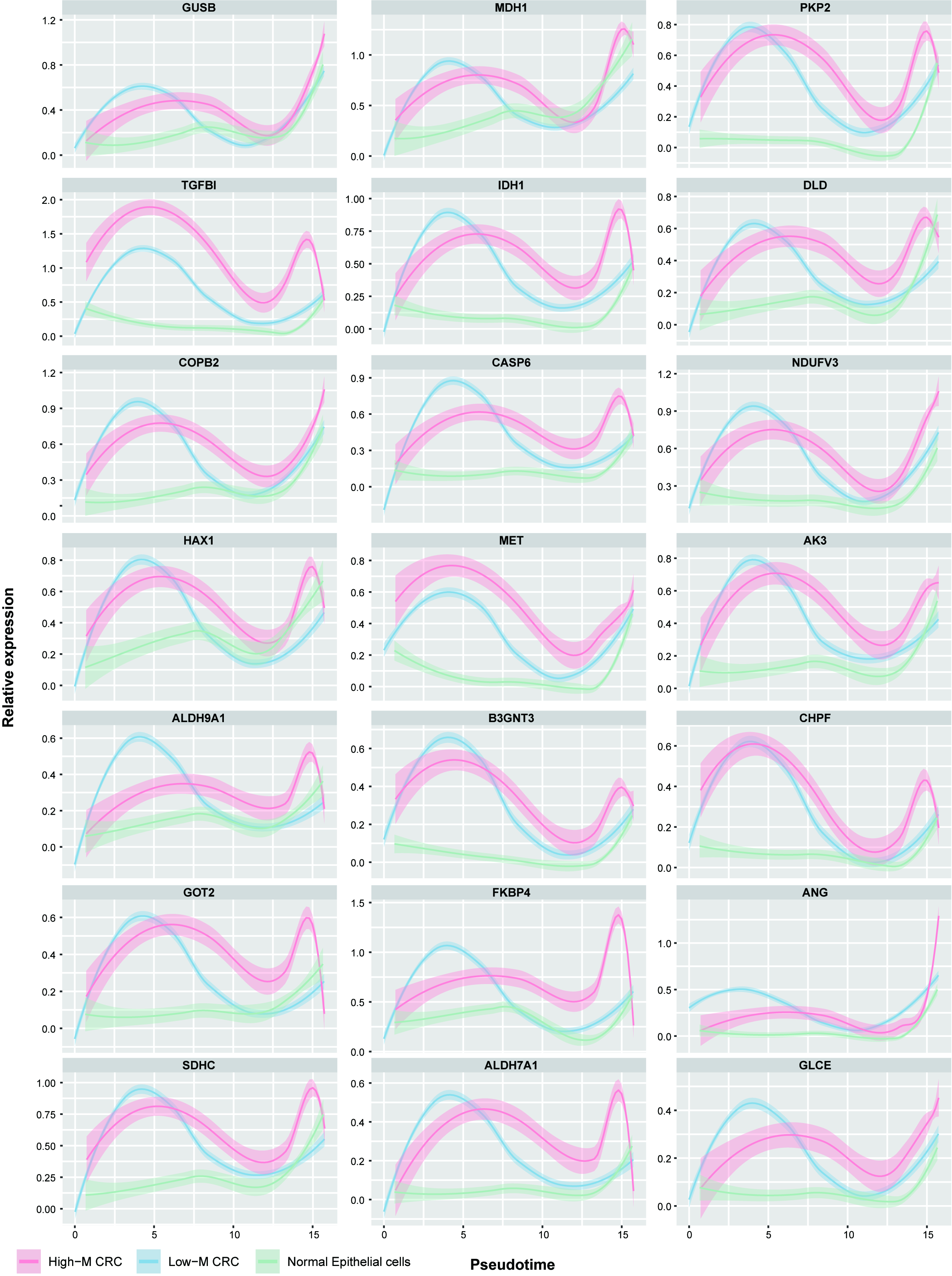

Supplement: Supplementary file 3 [file Image4.tif]

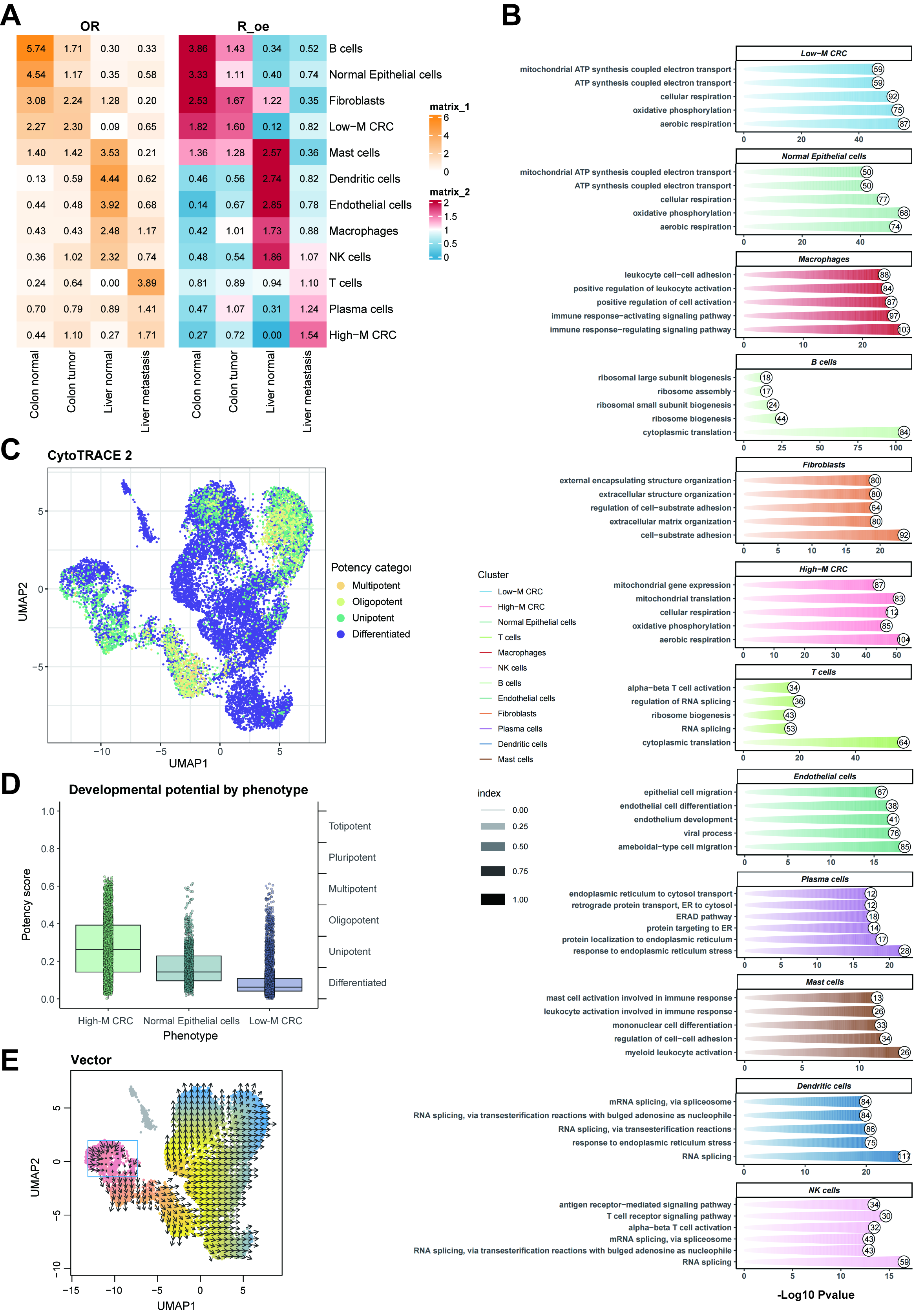

Supplement: Supplementary file 4 [file Image2.tif]

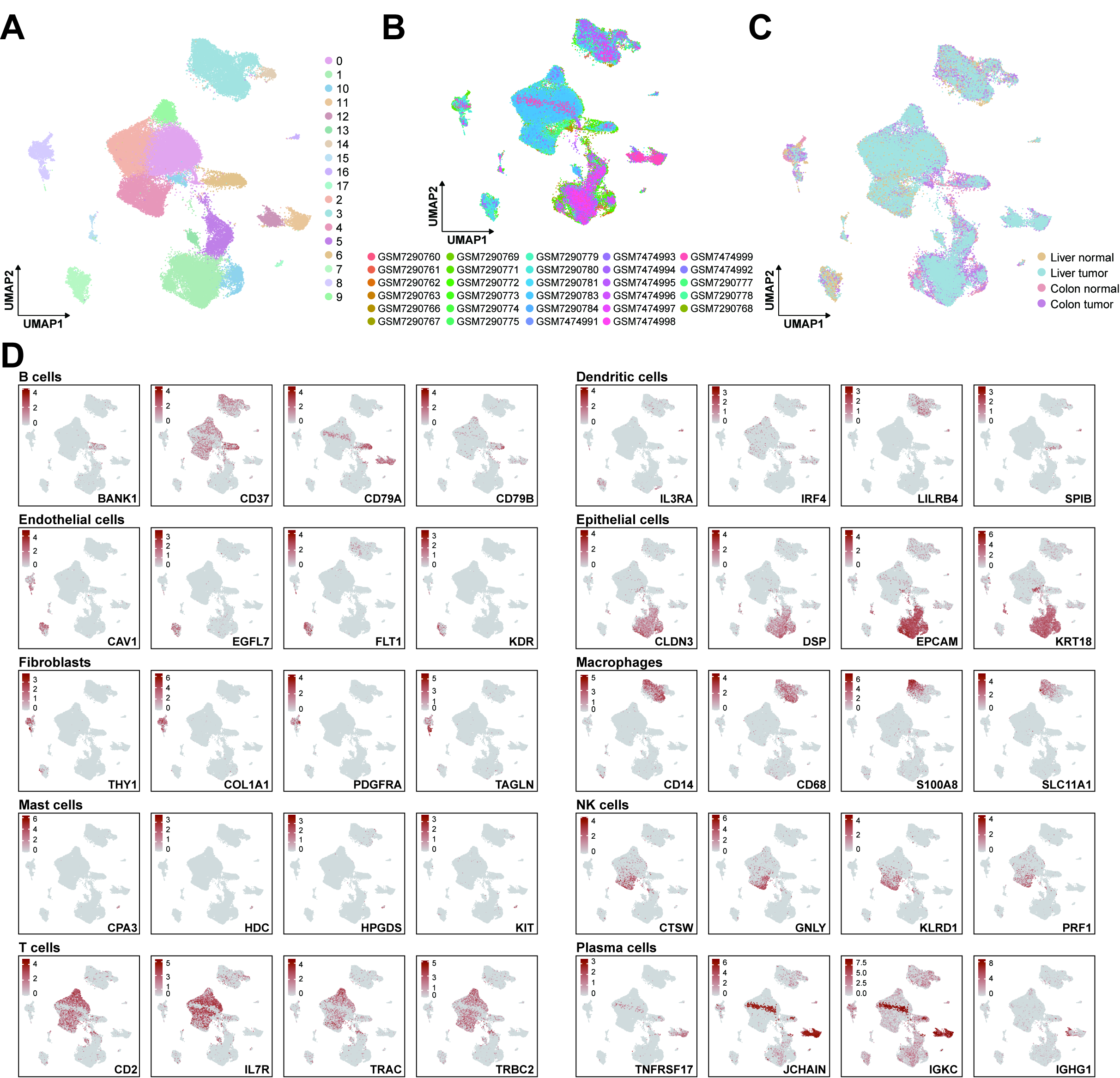

Supplement: Supplementary file 5 [file Image1.tif]

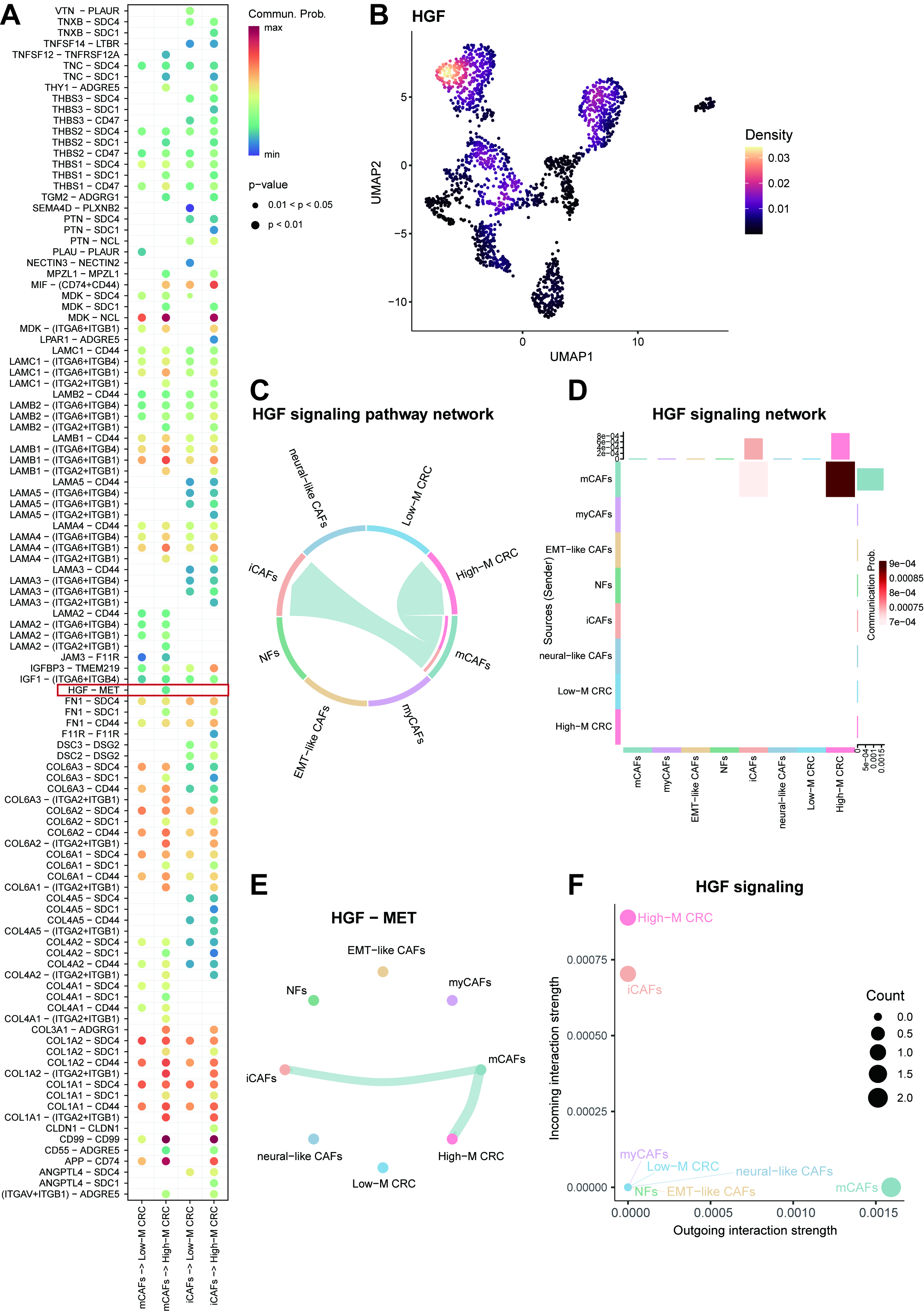

Supplement: Supplementary file 6 [file Image5.tif]
